# Long intergenic non-coding RNA induced by X-ray irradiation regulates DNA damage response signaling in the human bronchial epithelial BEAS-2B cell line

**DOI:** 10.3892/ol.2014.2622

**Published:** 2014-10-17

**Authors:** YANG JIAO, CHANG LIU, FENG-MEI CUI, JIA-YING XU, JIAN TONG, XIAO-FEI QI, LI-LI WANG, WEI ZHU

**Affiliations:** 1 Jiangsu Provincial Key Laboratory of Radiation Medicine and Protection, School of Radiation Medicine and Protection, Medical College of Soochow University, Suzhou, Jiangsu 215123, P.R. China; 2 School of Public Health, Medical College of Soochow University, Suzhou, Jiangsu 215123, P.R. China; 3 Department of Radiotherapy, The First Affiliated Hospital of Soochow University, Suzhou, Jiangsu 215006, P.R. China; 4 Department of Urology, The First Affiliated Hospital of Soochow University, Suzhou, Jiangsu 215006, P.R. China

**Keywords:** long non-coding RNA, radiation-induced lung injury, radiosensitivity, cell cycle, DNA damage response, p53

## Abstract

Long non-coding RNAs (lncRNAs) have been regarded as the primary genetic regulators of several important biological processes. However, the biological functions of lncRNAs in radiation-induced lung damage remain largely unknown. The present study aimed to investigate the potential effects of lncRNAs on radiation-induced lung injury (RILI). Female C57BL/6 mice were exposed to 12 Gy single doses of total body irradiation (TBI). LncRNA microarray screening was conducted at 24 h post-irradiation (IR) to investigate the differentially-expressed lncRNAs during RILI. Following the subsequent bioinformatics analysis and reverse transcription-polymerase chain reaction (RT-PCR) validation, one of the verified differentially-expressed long intergenic radiation-responsive ncRNAs (LIRRs), LIRR1, was selected for further functional study. The normal human bronchial epithelial BEAS-2B cell line was used as the cell model. The recombinant eukaryotic expression vector for the lncRNA was designed, constructed and transfected using lipofectamine. RT-PCR, clonogenic and flow cytometry assays, immunofluorescence detection and western blot analysis were performed to reveal the role of the lncRNA in the radiosensitivity regulation of the RILI target cells. In lung tissues 24 h after 12 Gy TBI, six of the identified differentially-expressed LIRRs near the coding genes were validated using quantitative (q)PCR. The upregulation of two LIRRs was observed and confirmed using qPCR. LIRR1 was chosen for further functional study. Following the stable transfection of LIRR1, identified through G418 screening, increased radiosensitivity, evident cell cycle G_1_ phase arrest and increased γ-H2AX foci formation were observed in the bronchial epithelial BEAS-2B cell line subsequent to IR. LIRR1 overexpression also led to decreased expression of the KU70, KU80 and RAD50 DNA repair proteins, marked activation of p53, decreased mouse double minute 2 homolog (MDM2) expression, and substantially induced p21 and suppressed cyclin-dependent kinase 2 in BEAS-2B following IR. Subsequent to the use of Pifithrin-α, a specific inhibitor of p53 activation, increased MDM2 expression was observed in the LIRR1-overexpressing cells, suggesting that LIRR1 could mediate the DNA damage response (DDR) signaling in a p53-dependent manner. The present study provides a novel mechanism for RILI, using the concept of lncRNAs.

## Introduction

Radiation-induced lung injury (RILI) is a common dose-limiting complication of thoracic radiotherapy that usually consists of radiation pneumonitis and radiation fibrosis (1–4). The occurrence of RILI is a continuous and dynamic process of damage-related signal transduction, amplification and feedback (1,2,5). However, the underlying molecular mechanisms and signaling pathways remain insufficiently understood, compromising effective intervention in RILI (3,5). Therefore, other potential mechanisms should be explored to integrate a more comprehensive and detailed strategy for the clinical prevention and treatment of RILI (4).

Long non-coding RNAs (lncRNAs) are a novel class of messenger RNA (mRNA)-like transcripts (6,7). In contrast to small non-coding RNAs, including microRNA and transcription initiation RNAs, lncRNAs are usually >200 nucleotides in length and lack an open reading frame (6–8). In the last decade, increasing evidence has established that, apart from microRNAs, lncRNAs may also be primary genetic regulators of several important biological processes, including metabolism, development and carcinogenesis (7,9). Although lncRNAs have previously been reported to conduct a broad spectrum of molecular and cellular roles by implementing different modes of action, including chromatin modification and epigenetic regulation, subcellular and structural organization of transcripts, and regulation of the expression of neighboring genes either in *cis* or *trans* form, a phenomenon known as transvection, their physiological functions remain poorly understood (6,7). Several studies have previously reported a group of lncRNAs that may be involved in the modulation of several cellular stress responses (10,11). For example, the increased expression of the psoriasis susceptibility-related RNA gene induced by stress (PRINS), a lncRNA gene, is induced by stress signals, including ultraviolet-B irradiation (IR), viral infection and translational inhibition (12). Certain large intergenic ncRNAs (lincRNAs) were also found to be regulated by the p53 pathway, which is involved in the DDR (13,14). However, in terms of the ionizing IR-induced DDR, the deregulation and biological functions of IR-responsive lncRNAs remain largely unknown.

IR can also induce stress signals, leading to systemic organ damage. Therefore, we hypothesized that lncRNAs may be involved in the process of RILI and may play a key modulatory role. In the present study, female C57BL/6 mice were used as an RILI model (15,16) and several differentially-expressed long intergenic radiation-responsive ncRNAs (LIRRs) were identified through microarray screening and quantitative polymerase chain reaction (qPCR) verification. Following bioinformatics analysis, LIRR1 was chosen for further functional study. A recombinant eukaryotic expression vector for LIRR1 was constructed and transfected into the normal human epithelial BEAS-2B cell line. Following RNA transcription-level confirmation by reverse transcription (RT)-PCR, the radiosensitivity of the BEAS-2B cells constantly overexpressing LIRR1 was assessed through clonogenic and flow cytometry assays. The IR-induced DNA double-strand breaks (DSBs) were visualized by immunofluorescence staining. The associated mechanism was preliminarily revealed by western blot analysis. The present results may provide novel mechanisms that result in the occurrence and development of RILI *in vitro*.

## Materials and methods

### Animals

C57BL/6 mice (8–9 weeks old; Slac Laboratory Animal Co., Ltd., Shanghai, China) were used in the present study. All the mice were housed and maintained in a mouse colony, with a maximum of six mice per cage. All the animal procedures were conducted in accordance with the regulations of the Animal Experimentation Ethics Committee of Soochow University (Suzhou, Jiangsu, China).

### Radiation exposure

The mice were anesthetized with 50 mg/kg pentobarbital sodium (Sigma-Aldrich, St. Louis, MO, USA) through intraperitoneal injection prior to IR. An immobilization chamber allowing simultaneous bilateral exposure was used for total body IR (TBI). Using a 6-MeV electron beam accelerator (Siemens KD-2; Siemens, Munich, Bavaria, Germany) at a dose rate of 200 cGy/min and with a source to skin distance of 100 cm, 12 Gy was delivered to the mid-plane. Thermoluminescence dosimeters were placed over the selected mice to verify the administration of the correct dose for quality assurance. For cultured cell exposure, an IR area of 20×20 cm and a source to skin distance of 100 cm were used, and doses of 0, 0.5, 1, 2, 4 and 6 Gy were delivered separately to the mid-plane.

### Microarray and computational analysis

The lungs of the mice in the sham-irradiated and 12 Gy TBI groups were homogenized in TRIzol (Invitrogen, Carlsbad, CA, USA). Total RNA was isolated using a Qiagen RNase Mini kit (Qiagen, Valencia, CA, USA). The RNA concentration and integrity were measured using a NanoDrop ND-100 spectrophotometer (Thermo Fisher Scientific, Inc., Waltham, MA, USA) and denaturing agarose gel electrophoresis, respectively.

The mouse lncRNA microarray analysis was performed by KangChen Bio-tech (Shanghai, China). Briefly, mouse lung tissue mRNA was purified using a mRNA-ONLY™ Eukaryotic mRNA Isolation kit (Epicentre Biotechnologies, Madison, WI, USA) according to the manufacturer’s instructions. An Agilent array platform (Agilent Technologies, Inc., Santa Clara, CA, USA) was used for the subsequent analysis. One-color fluorescence-labeled complementary RNA, transcribed without 3′ bias, was prepared using a Quick Amp labeling kit and hybridized with the Mouse lncRNA Microarray v2.0 (8×60 K; ArrayStar, Rockville, MA, USA), which contained 31,423 lncRNAs and 25,376 coding transcripts that were carefully collected from the most authoritative databases, such as RefSeq (National Center for Biotechnology Information, Bethesda, USA), UCSC Knowngenes (University of California, Santa Cruz, CA, USA), and Ensembl (Hinxton, Cambridgeshire, UK). Each treatment was performed in triplicate, with three array sets being prepared for the sham and irradiated lung tissues.

An Agilent Technologies G2505B Scanner was used for array scanning, and Agilent Feature Extraction software v11.0.1.1 was used to acquire the array images required for the data analysis (both Agilent Technologies, Inc.). Quantile normalization and subsequent data processing were conducted using the GeneSpring GX v11.5.1 software package (Agilent Technologies, Inc.).

### qPCR

Total RNA was extracted with TRIzol and reverse-transcribed into complementary DNA (cDNA) using the oligo(dT)_18_ primer and HiscriptTM First-strand cDNA Synthesis kit (Vazyme Biotech (Nanjing) Co., Ltd, Nanjing, Jiangsu, China). The transcription levels of LIRR1 and β-actin, the control gene, were determined using the SYBR green method (Applied Biosystems 7500FAST RT-PCR system; Applied Biosystems, Foster City, CA, USA). The primers were designed using Primer3 software (available from bioinfo.ut.ee/primer3; Table I). Each PCR used 0.2 μl platinum Taq polymerase, 0.5 μl of each PCR primer (10 mM), 1 μl SYBR (20x), 1 μl cDNA, 2 μl 10X PCR buffer, 2 μl Mg^2+^ (25 mM), 0.5 μl deoxynucleotide triphosphates (25 mM) and 20 μl double-distilled water. For the RT-PCR, the denaturation step was established at 95°C for 2 min and then continued at 95°C for 10 sec, 60°C for 30 sec and 70°C for 30 sec for 40 cycles, with a final extension step at 70°C for 8 min. All the RNA samples were analyzed in triplicate for each tested transcript and the levels were normalized to β-actin. Expression fold changes were calculated using the 2^−ΔΔCt^ method (10).

### Cell culture and transfection

The human bronchial epithelial BEAS-2B cells were originally obtained from the Type Culture Collection (Chinese Academy of Sciences, Shanghai, China) and maintained at Jiangsu Provincial Key Laboratory of Radiation Medicine and Protection (Suzhou, China). The cells were cultured with Dulbecco’s modified Eagle’s medium (Invitrogen) supplemented with 10% fetal bovine serum (Invitrogen, Carlsbad, CA, USA) at 37°C in an atmosphere of 5% CO_2_. The medium was changed every two days, and the cells were sub-cultured every three days.

The recombinant plasmid pcDNA3.1/LIRR1 was constructed by Jiangsu Provincial Key Laboratory of Radiation Medicine and Protection as previously described (data not shown) (17). The plasmid transfection was conducted using lipofectamine (Invitrogen), as previously described (18). G418 (500 μg/ml; Invitrogen, Carlsbad, CA, USA) was used to acquire resistant clones with high LIRR1 transcription. The LIRR1 transcription was determined using an RT-PCR assay. the denaturation step was established at 95°C for 2 min and then continued at 95°C for 10 sec, 60°C for 30 sec and 70°C for 30 sec for 40 cycles, with a final extension step at 70°C for 8 min.

### Clonogenic assay

The exponential cells were plated at various cell densities and irradiated with 0, 0.5, 1, 2, 4 and 6 Gy of X-ray radiation. Following 12–14 days of incubation at 37°C, the cells were fixed in methanol, which was followed by Giemsa staining. The number of colonies per dish was counted and the surviving fractions were calculated as the ratio of plating efficiencies for irradiated and non-irradiated cells. Plating efficiency was defined as the colony number divided by the number of cells plated for the non-irradiated controls. The experiments were conducted in triplicate and the data are presented as the mean ± standard deviation from three independent experiments. All the survival fractions were plotted onto a linear quadratic model using GraphPad Prism 5.0 software (GraphPad Software Inc., La Jolla, CA, USA).

### Flow cytometry

The cells were removed with trypsin and collected in centrifuge tubes together with the culture medium. The detailed method for flow cytometry has been previously described (17). The cell suspensions were collected and centrifuged at 1,800 × g for 5 min. The supernatant was discarded, and the cell pellets were washed with 1X phosphate-buffered saline (PBS, Hyclone, Logan, UT, USA) and centrifuged at 1,800 × g for 5 min. Finally, the cells were fixed in 5 ml chilled 70% ethanol at 4°C for 4 h. Following centrifugation and washing with 1× PBS three times, the cell pellets were resuspended in 500 μl propidium iodine (10 μg/ml) containing 300 μg/ml RNase (Sigma-Aldrich). The cells were then incubated on ice for 30 min and filtered with a 53 μm nylon mesh. The cell cycle distribution was calculated from 10,000 cells using ModFit LT software (Becton Dickinson, San Jose, CA, USA) and FACSCalibur (Becton Dickinson).

### Immunofluorescence assay

The cells were seeded on a sterile glass chamber (Becton Dickinson, Franklin Lakes, NJ, USA) at the appropriate density and incubated overnight at 37°C in an atmosphere of 5% CO_2_. Following exposure to 4 Gy X-ray IR for 1 h, the cells were fixed using 4% formaldehyde for 30 min and then permeabilized using 0.25% Triton X-100. The 1% bovine serum albumin was used for blocking for 1 h at room temperature. The primary rabbit anti-human monoclonal anti-phosphorylation γ-H2AX antibody (1:1,000; Epitomics, Burlingame, CA, USA) was used for a 2-h incubation at room temperature. A specific monoclonal goat anti-rabbit secondary antibody conjugated to CY3 (1:2,000; Beyotime Institute of Biotechnology, Haimen, China) was incubated in the dark for 1 h at room temperature, followed by 5 μg/ml Hoechst 33342 nuclear staining (Sigma-Aldrich). Fluorescence was observed using laser scanning confocal microscopy at a wavelength of 650 nm within 30 min. The number of foci in 50 cells in each sample was counted randomly.

### Western blot assay

Western blot analysis was performed as previously described (17). Briefly, the following primary antibodies were used for immunoblotting: RAD50 (catalog number 3427), KU70 (clone, D10A7), KU80 (clone, C48E7), p-p53 (Ser15; clone, D4S1H), p21 (clone, 12D1) (all 1:1,000 dilution; Cell Signaling Technology, Danvers, MA, USA), cyclin-dependent kinase 2 (CDK2; clone, M2; dilution, 1:1,000; Santa Cruz Biotech, CA, USA), mouse double minute 2 homolog (MDM2; clone, S1357; dilution, 1:1,000; Epitomics Inc., Hangzhou, Zhejiang, China) and β-actin (clone, C4; dilution, 1:1,000; Santa Cruz Biotechnology, Inc., Dallas, TX, USA). The appropriate secondary antibodies (1:5,000; Bioworld Technology Inc., St. Louis Park, MN, USA), enhanced chemiluminescence system (Union Bioscience Corporation, Hangzhou, Zhejiang, China), and X-film (Carestream Health, Shanghai, China) were used for developing the immunoblot using pre-stained markers (catalog number 26616; Thermo Fisher Scientific, Inc.) as the molecular size standard.

### Statistical analysis

The data are presented as the mean ± standard deviation. The statistical comparisons of the experimental results between the treated and control groups were performed using the two-tailed Student’s t-test. All statistical tests were measured using SPSS version 17.0 (SPSS Inc., Chicago, IL, USA). P<0.05 was considered to indicate a statistically significant difference.

## Results

### Screening, validation and analysis of differentially-expressed lincRNA LIRR1 from the RILI mouse model

**Figure 1:**
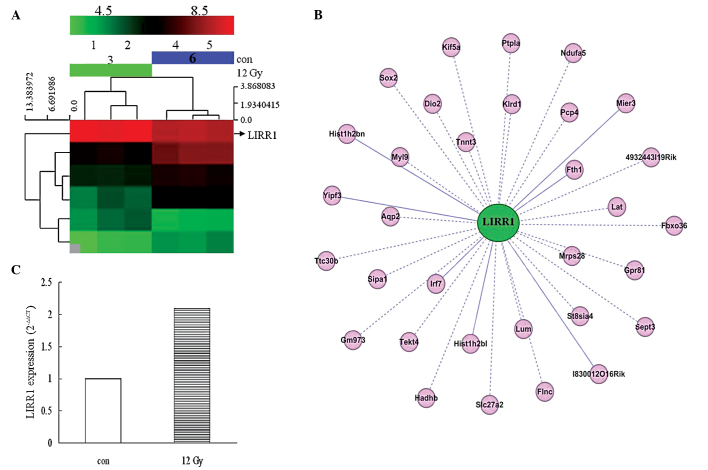
Screening, validation and analysis of differentially-expressed large intergenic non-coding RNA-LIRR1 in lung tissue with acute radiation-induced lung injury. (A) Hierarchical cluster analysis diagram of 12-Gy X-ray irradiated lung tissues (n=3) vs. normal lungs (n=3). The arrow pointing to the LIRR1 gene and the color saturation varying from green to red represent the log2 value of the normalized intensity of each sample, ranging between 4.5 and 8.5 (P-value limit, 0.05). The rows indicate the individual probe sets, and the columns denote the experimental sample. (B) Co-expression network of LIRR1 and the encoding genes. The R-value was calculated to define the correlation coefficient, PCC, between LIRR1 and the encoding genes. A value of ≥0.99 was considered to indicate a co-expression pair. CytoScape software was used to integrate the interaction network between LIRR1 and the encoding genes. The pink nodes indicate the encoding genes and the green node denotes LIRR1. A solid line between two nodes indicates a positive correlation and a dotted line indicates a negative correlation. (C) LIRR1 transcription was validated in normal mouse lung tissues (white bar) and lung tissues from mice exposed to total body irradiation for 24 h (striped column; n=3 for each treatment). Reverse-transcription polymerase chain reaction was conducted in triplicate for each individual sample. All the experiments were performed independently at least three times. LIRR, long intergenic radiation-responsive non-coding RNA; con, control.

Six differentially-expressed lincRNAs that were near coding genes (distance, <300 kb) and whose expression was significantly different between the two groups (TBI vs. normal, log fold change≥2.0; P≤0.05) were identified. As shown in the hierarchical clustering dendrogram, all six lung tissue samples were arranged into two groups, TBI vs. normal control, and the results revealed a distinguishable lincRNA expression profile (Fig. 1A). The six lncRNAs were divided into two groups based on their response to IR. Group I included LIRR1 and 5, which were induced following 24 h of X-ray exposure, and group II included LIRR2, 3, 4 and 6, which decreased following exposure.

The 273-bp X-ray inducible lincRNA LIRR1, which was located on the positive strand of chromosome 1 and was adjacent to the Stat4 gene (distance, <300 bp), was classified as a sense overlap lncRNA. This type of lncRNA is able to exert biological effects through gene transcription and expression regulation (19). As shown in Fig. 1B, further bioinformatics analysis revealed the co-expression pattern of LIRR1 with 32 coding genes, which are mainly associated with cell cycle regulation (Sept3, Sipal and Sox2), chromatin structure (Hist1h2b1, Hist1h2b1n and Mier3), immune response (Klrd1, Lat and Irf7), mitochondrial structure (Ndufa5, Hadhb, and Mrps28), collagen fibril organization and circumferential growth, corneal transparency and epithelial cell migration and tissue repair (Lum) (Fig. 1B). The induced transcription of LIRR1 in TBI mouse lung tissues was also validated by qPCR (Fig. 1C). Thus, LIRR1 was selected for further study. The sequence of LIRR1 was acquired from the UCSC Genome Browser database (http://genome.ucsc.edu/) according to the microarray screening results.

### LIRR1 regulates the radiosensitivity of human bronchial epithelial BEAS-2B cells to X-ray IR

**Figure 2:**
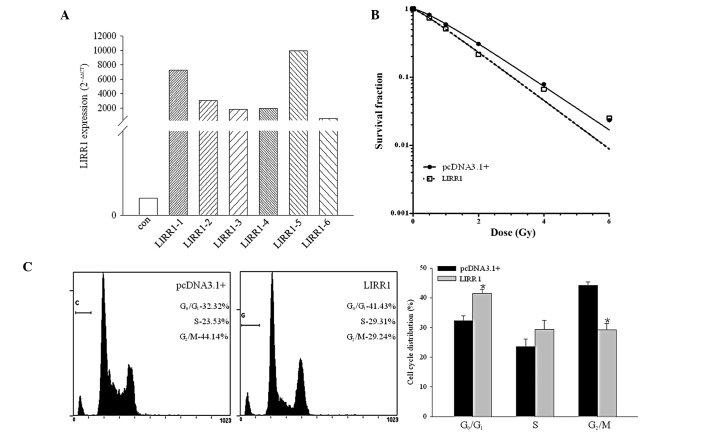
High LIRR1-transcription radiosensitivity of human bronchial epithelial BEAS-2B cells to X-ray radiation. (A) The cells transfected with LIRR1 recombinant plasmid, were selected by 500 μg/ml G418 screening. Resistant clones were amplified and further assessed by reverse transcription-polymerase chain reaction to determine the LIRR1 tanscription level. (B) The cells were irradiated with a single dose of X-ray radiation at 0.5, 1, 2, 4 or 6 Gy. Colonies >50 cells were counted and assessed by Giemsa staining ≥10 days after IR. (C) At 24 h post-exposure to X-ray radiation, the cells were collected, fixed with cold 70% ethanol and stained with propidium-iodide. Flow cytometry was performed, with each sample prepared in triplicate. The cell cycle distribution was compared using a two-tailed t-test between the control and LIRR1-overexpressing groups. Results are representative of three individual experiments. LIRR, long intergenic radiation-responsive non-coding RNA; con, control.

In the present study, the transcription level of LIRR1 was measured by RT-PCR, as shown in Fig. 2A. The transcription of the six selected resistant clones, which were stably transfected with the pcDNA3.1/LIRR1 plasmid, was increased significantly compared with that of the parental BEAS-2B cells. Clone 5 was identified for the following studies due to the highest transcription level of LIRR1. At the same time, six clones that were stably transfected with the pcDNA3.1 vector were also assessed using RT-PCR assay to determine their LIRR1 transcription level (data not shown). One of the six resistant clones exhibited a similar transcription pattern to the parental BEAS-2B cells and was therefore also selected for the following study.

LncRNA LIRR1 markedly affected the radiosensitivity of the BEAS-2B cells. As shown in Fig. 2B, the mock transfectant, BEAS-2B/pc3.1, revealed a typical clonogenic survival curve with a shoulder, indicating the potential of DNA damage repair. Exogenous LIRR1 significantly radiosensitized the BEAS-2B cells to X-ray IR.

Flow cytometry was performed 24 h after the cells were exposed to 4 Gy of X-ray IR. As shown in Fig. 2C, the LIRR1 transfectant cells exhibited a significantly increased proportion of cells in the G_1_ phase (P<0.05) compared with the mock BEAS-2B/pc3.1 transfectant cells following IR.

### LIRR1 regulates γ-H2AX focus formation in BEAS-2B cells following IR

**Figure 3:**
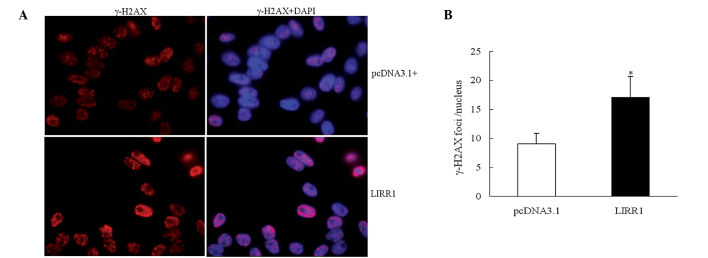
LIRR1 regulates γ-H2AX focus formation following IR. (A) LIRR1-overexpressing BEAS-2B cells and the BEAS-2B cells stably transfected with the vector were irradiated with a single dose of 4 Gy X-ray radiation followed by immunofluorescence within 1 h of IR. (B) Quantitative analysis of γ-H2AX foci per nucleus for 50 cells per data point. All the experiments were performed independently at least three times. ^*^P<0.05 vs. control group. LIRR, long intergenic radiation-responsive non-coding RNA; IR, irradiation.

The IR-induced phospho-γ-H2AX foci formation was regarded as the initial DDR, activating different cascades and leading to different outcomes (20,21). In the present study, immunofluorescence staining of phospho-γ-H2AX was visualized 1 h after X-ray IR to investigate the potential functions of LIRR1 on the IR-induced DDR. As shown in Fig. 3, there were approximately nine IR-induced γ-H2AX foci per nucleus in the pcDNA3.1 transfectants. Significantly increased numbers of γ-H2AX foci were observed in the BEAS-2B cells exhibiting a high LIRR1 transcription level, with ~17 per nucleus (t=−14.46; P<0.01). The present results suggest that lincRNA LIRR1 is able to inhibit IR-induced DNA damage repair.

### LIRR1 is involved in DSB damage signaling pathway regulation following IR in vitro

**Figure 4:**
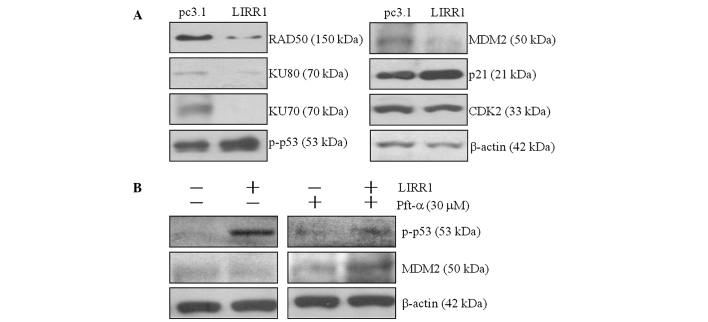
LIRR1 was involved in the regulation of the DDR signaling pathway after IR. (A) Western blot analyses were performed to determine the expression of DDR sensors and the downstream cascades in LIRR1-overexpressing BEAS-2B cells. The cells were exposed to 4 Gy of irradiation, and 50 μg of protein was assessed using SDS-PAGE. Immunoblotting with the indicated specific antibodies of the DDR signaling pathway was subsequently conducted. The bands were analyzed through densitometry, with β-actin as the loading control. (B) Following IR, the cells were cultured in the presence or absence of the p53 inhibitor Pft-α. The expression levels of p-p53 and MDM2 were determined by western blot analysis. A representative result was gained following three independent experiments. LIRR, long intergenic radiation-responsive non-coding RNA; DDR, DNA damage response; IR, irradiation; p-p53, phospho-p53; MDM2, mouse double minute 2 homolog; Pft-α, Pifithrin-α; CDK2, cyclin-dependent kinase 2.

Western blot analysis was performed to verify the mechanisms of LIRR1 modulating radiosensitivity. As shown in Fig. 4A, when exposed to X-ray radiation, the high transcription of LIRR1 led to the decreased expression of DSB sensors KU70 and KU80, DNA damage repair protein RAD50, cell cycle G_1_ phase checkpoint CDK2 and p53 partner MDM2 compared with the control group. The increased expression of the key regulator of DDR-phosphorylated p53 and p21 was observed due to the high transcription level of LIRR1. The present results are not only in accordance with the data drawn from the clonogenic assay, flow cytometry and immunofluorescence staining, but are also consistent with the bioinformatics analysis of the co-expression pattern of LIRR1 (Fig. 1B).

Phosphorylation and the subsequent activation of p53 and its transcriptional induction are the key initial responses to cell cycle distribution and DNA damage (21–24). In the present study, it was identified that the high transcription level of LIRR1 was coordinated with IR-induced p53 phosphorylation. Following the use of Pifithrin-α, a specific inhibitor of p53 activation, the decreased MDM2 expression in LIRR1-overexpressing cells following IR was restored, suggesting that LIRR1 could mediate the DDR signaling in a p53-dependent manner (Fig. 4B).

## Discussion

Guttman *et al* (8) demonstrated that lincRNAs are regulated during their development and in response to diverse signaling cues, including DNA damage, which may affect various human diseases (19). In total, >3,000 human lincRNA cases have been identified, of which, <1% have been characterized (14). The differentially-expressed lincRNAs associated with RILI have not been previously reported. In the present study, the differentially-expressed lincRNA was identified in C57BL/6 mouse lung tissue 24 h after TBI using microarray screening and RT-PCR validation. The expression of the six differential lincRNAs either increased, LIRR1 and 5, or decreased, LIRR 2 3, 4 and 6, in the irradiated mice compared with their sham-irradiated counterparts.

LIRR1 was chosen for further functional study due to the following reasons (1): LIRR1 was induced by X-ray IR, which may induce a variety of coding genes involved in the intracellular metabolic pathways and play an important role in cell cycle regulation (involving the GADD family, MDM2, WAF1 and CDK) apoptosis (involving the Bcl-2 family death receptors and caspase family), DNA damage repair (involving ATM, p53, Ras, PKC and ErbB) and cell growth regulation (involving EGFR and IGF-1) (20,21,24,25–29). However, the biological function and associated mechanisms of IR-induced lincRNAs remain largely unknown (2). The 273-nt lincRNA locus on chromosome 1 was co-expressed with 27 coding genes (Fig. 1B), which are mainly associated with cell cycle regulation (Sept3, Sipal and Sox2), chromatin structure (Hist1h2b1, Hist1h2b1n and Mier3), immune response (Klrd1, Lat and Irf7), mitochondrial structure (Ndufa5, Hadhb and Mrps28), collagen fibril organization and circumferential growth, corneal transparency, and epithelial cell migration and tissue repair (Lum).

The IR-induced DDR consists of several highly complex and coordinated signaling pathways, including programmed cell death, cell cycle regulation and DNA repair pathways, with each exerting different effects on cells (21,23). In the present study, a gain-of-function strategy was used, and the effect of LIRR1 on cell survival following IR was investigated through a clonogenic assay. LIRR1 overexpression significantly reduced clonogenic formation following IR and therefore, LIRR1 facilitated cell death following IR.

Another important pathway of DDR is the activation of cell-cycle checkpoints (23,24). Treatment of mammalian cells with IR causes delays in the movement of cells through the G_1_, S and G_2_ phases. In the present study, LIRR1-overexpressing BEAS-2B cells exhibited a significant increase in the G_1_ phase distribution of the cell cycle following IR.

As one of the earliest occurring events, the phosphorylation of H2AX, also known as γ-H2AX, is necessary for the recruitment of other proteins involved in DDR (30,31). Cells lacking H2AX exhibit a substantial increase in radiosensitivity (20). BEAS-2B cells with a high LIRR1 transcription level produce a higher amount of γ-H2AX foci following IR than the ‘mock’ constantly transfected control cells. Along with the results of the clonogenic assay, the phenotype of increased radiosensitivity may reflect the potential regulatory effects exerted by LIRR1 on the associated regulatory factors of DDR.

Previous studies have revealed that hundreds of proteins can be phosphorylated by two key kinases, ataxia telangiectasia mutated (ATM) and ataxia telangiectasia and Rad3-related (ATR), in response to DNA damage (22,28). Therefore, these kinases serve as the signals to activate several downstream effectors of the DDR, including DNA repair, cell cycle checkpoints and apoptosis. One of the most important proteins activated by ATM and ATR is p53 (24,29). Once activated by DSB-induced ATM phosphorylation, the stabilization of the p53-MDM2 complex is disturbed (29). Consequently, the p53 protein is no longer degraded. A series of genes is induced by activated p53, which affects the checkpoints or promotes apoptosis. The induction of the p53 downstream effector, p21, inhibits the function of cyclin-CDKs complex and interrupts cell entry into the S phase (29).

The present study reports the evident decrease of DNA repair protein KU70, KU80 and RAD 50 in LIRR1-overexpressing BEAS-2B cells following IR and the marked activation of p53, the decrease in MDM2, the substantial inducement of p21 and the suppression of CDK2. These findings may explain the resulting block of the cell cycle checkpoint and DNA damage repair. In accordance with this finding, the control cells clearly did not demonstrate the same expression pattern as the aforementioned proteins. Furthermore, Pifithrin-α was used following IR. The ability of this p53 inhibitor to affect MDM2 expression indicates the direct role of p53 in modulating the radiation response in LIRR1-overexpressing BEAS-2B cells. The present findings suggest a potential biological role of lincRNAs in RILI, which is considered the most common clinical complication of thoracic radiotherapy. However, the molecular basis of such a role remains unclear.

In conclusion, according to the present preliminary study, varying lincRNAs expression may be involved in the regulation of the RILI process and can be profiled to aid the diagnosis, prognosis and even therapy of RILI. LIRR1 may be a novel molecular target of IR, as it was able to modulate the radiosensitivity of BEAS-2B cells through the DDR signaling pathways in a p53-dependent manner. However, several questions remain unanswered (1). It should be determined whether the variable lincRNAs identified in the present study respond only to IR or not, as a group of long stress-responsive non-coding transcripts, including Xist and HOTAIR, has already been identified (2). Although the biological functions of certain long IR-induced non-coding RNAs were initially explored, the precise roles and mechanisms of LIRR-ncRNAs in RILI development mentioned in the present study necessitate further *in vivo* and *in vitro* experimental investigation.

## Figures and Tables

**Table I:** Primers for quantitative polymerase chain reaction.

Gene name	Forward	Reverse	Product length, bp
β-actin	CACTATTGGCAACGAGCGGT	CAACGTCACACTTCATGATGGA	119
LIRR 1	CATGGTGGCTCACAATTATCTGTAA	CATATATGTCAGTACACCATCACTGTCTTC	85
LIRR 2	CAACTTTTCCCTCGGGATTATAAC	AGTACCCAAGGTTGAAGCCATAAA	100
LIRR 3	ACTCCAGTGACCTGCATGCA	AGTGTGAAAGGCTCTGTGGGTTA	76
LIRR 4	TGGTCAGAACCCTTGCTATTTTATT	AGAGGGCACTTCAGCCAAGTT	75
LIRR 5	CCGGGAGACATTGGCACTT	AAGTTGACTTTGAACTGTGCCATATG	82
LIRR 6	AAAGCGGCAGCAGATTGC	TGCCAGTAGGGTAAAGCAGTTCT	81
LIRR 7	TCTTCATTTCAGAGGGACAGCTT	CTGTTGCAGAGCCATGAAGGT	88
LIRR 8	GGCAAGCTCTATGGCAACGA	CATGTTCTTGAAGCTCAGGTTGA	77
LIRR 9	AGTTTTCAGCTATTGATAAACTTGGACA	AAAAGTTTCCATTTTTTGGTGAGTAGA	97

LIRR, long intergenic radiation-responsive non-coding RNA.

## Abbreviations

lncRNA: long non-coding RNA
lincRNA: long intergenic non-coding RNA
RILI: radiation-induced lung injury
TBI: total body irradiation
RT-PCR: reverse transcription-polymerase chain reaction
qPCR: quantitative polymerase chain reaction
